# Risk factors for residual back pain following percutaneous vertebral augmentation: the importance of paraspinal muscle fatty degeneration

**DOI:** 10.1007/s00264-023-05809-7

**Published:** 2023-04-19

**Authors:** Xiangcheng Gao, Jinpeng Du, Dingjun Hao, Baorong He, Liang Yan

**Affiliations:** 1 Department of Spine Surgery, Honghui Hospital, Xi’an Jiaotong University, Youyidong Road 555, Xi’an City, Shaanxi Province China; 2 Yan’an University, Yan’an City, Shaanxi Province China

**Keywords:** Osteoporosis, Percutaneous vertebral augmentation, Residual back pain, Paraspinal muscle fatty degeneration

## Abstract

**Purpose:**

Residual back pain (RBP) after percutaneous vertebral augmentation (PVA) still exists considerable, and it even affects daily life due to moderate or severe back pain. A variety of risk factors have been previously identified for developing residual back pain. However, there are conflicting reports regarding the association between sarcopenia and residual back pain. As such, the aim of this study was to investigate whether paraspinal muscle fatty degeneration is a predictor of residual back pain.

**Methods:**

We retrospectively reviewed the medical records of patients with single-segment OVCF who underwent PVA from January 2016 to January 2022. Patients were divided into RBP group (86 patients) and control group (790 patients) according to whether the visual analog scale (VAS) score ≥ 4. The clinical and radiological data were analyzed. Paraspinal musculature fatty degeneration was measured using the Goutallier classification system (GCS) at the L4 − 5 intervertebral disc level. Univariate and multivariate logistic regression analyses were performed to identify risk factors.

**Results:**

The results of multivariate logistical regression analysis revealed that posterior fascia injury (odds ratio (OR) = 5.23; 95% confidence interval (CI) 3.12–5.50; *P* < 0.001), as regards paraspinal muscle fatty degeneration, including Goutallier grading (*OR* = 12.23; 95% *CI* 7.81–23.41; *P* < 0.001), fCSA (*OR* = 3.06; 95% *CI* 1.63–6.84; *P* = 0.002), fCSA/CSA (%) (*OR* = 14.38; 95% *CI* 8.80–26.29; *P* < 0.001), and facet joint violation (*OR* = 8.54; 95% *CI* 6.35–15.71; *P* < 0.001) were identified as independent risk factors for RBP.

**Conclusions:**

Posterior fascia injury, paraspinal muscle fatty degeneration, and facet joint violation were identified as independent risk factors for RBP, with paraspinal muscle fatty degeneration playing an important role.

## Introduction

Osteoporotic vertebral compression fracture (OVCF) is becoming a common source of back pain and progressive spinal deformity, reducing quality of life and becoming an increasingly serious health problem worldwide [1]. Treatment for OVCF includes conservative and surgical approaches. Conservative treatment of OVCF is with pain medications, stability interventions, physical braces, and bed rest [2]. Percutaneous vertebral augmentation (PVA) is the standard surgical operation for OVCF, including percutaneous vertebroplasty (PVP) and percutaneous kyphoplasty (PKP), which mainly involves injecting polymethyl methacrylate (PMMA) bone cement into the collapsed vertebral bodies to relieve fracture-related pain and enhance the stability and strength of vertebral bodies [3, 4].

However, several scholars were still skeptical about the analgesic effect of PVA [5, 6]. Residual back pain (RBP) still exists in a proportion of patients after PVA, and it even affects daily life due to moderate or severe back pain. It has been investigated that the proportion of unrelieved back pain after PVA is about 5 to 20% [7]. Previously, various factors have been considered to be involved in the persistence of RBP after PVA, including nonunion, bone density, bone cement volume, bone cement distribution, and thoracolumbar dorsal fascia injury [8–10]. However, there are fewer related studies and the conclusions are somewhat controversial. In contrast, paraspinal muscle fatty degeneration was confirmed to be an important risk factor for the development of chronic low back pain [11]. We thought that along with fractured body-related factors, the paraspinal muscle also affects the surgical outcome and quality of life. To the best of our knowledge, no prior studies have evaluated the impact of paraspinal muscle fatty degeneration on the development of RBP following PVA using opportunistic evaluation of paraspinal fatty degeneration on preoperative magnetic resonance imaging (MRI). Therefore, we conducted this study to analyze whether paravertebral muscle fatty degeneration is a primary risk factor for RBP.

## Methods

### General data

This retrospective study analyzed 876 patients with OVCF who underwent PVA in the authors’ institution between from January 2016 and January 2022. This study protocol was approved by the Medical Ethics Committee of our Hospital, and all patients obtained preoperative informed consent.

Inclusion criteria were as follows: (1) patients diagnosed with OVCF; (2) treated with PVA; (3) follow-up period of more than 12 months.

Exclusion criteria were as follows: (1) defined cement leakage into spinal canal, (2) patients with neurological deficits, (3) new postoperative vertebral fracture or postoperative infection, (4) combined with malignant tumors of the spine, (5) combined with severe cardiac and pulmonary dysfunction, unable to tolerate surgery.

### Evaluation index

The following clinical and radiographic data were recorded: demographic data (age, gender, body mass index (BMI)), comorbidities (hypertension, diabetes, chronic obstructive pulmonary disease (COPD) and others), preoperative VAS, preoperative imaging data (bone mineral density (BMD), injured vertebral segment, vertebral height loss, local kyphosis angle, intravertebral vacuum cleft, posterior fascia injury and paraspinal muscle fatty degeneration), surgical data (surgical method, surgical approach, cement viscosity, cement volume), postoperative radiological parameters (vertebral height restoration, cement distribution, facet joint violation and cement leakage) and postoperative bracing treatment.

### Index definition

**Fig. 1:**
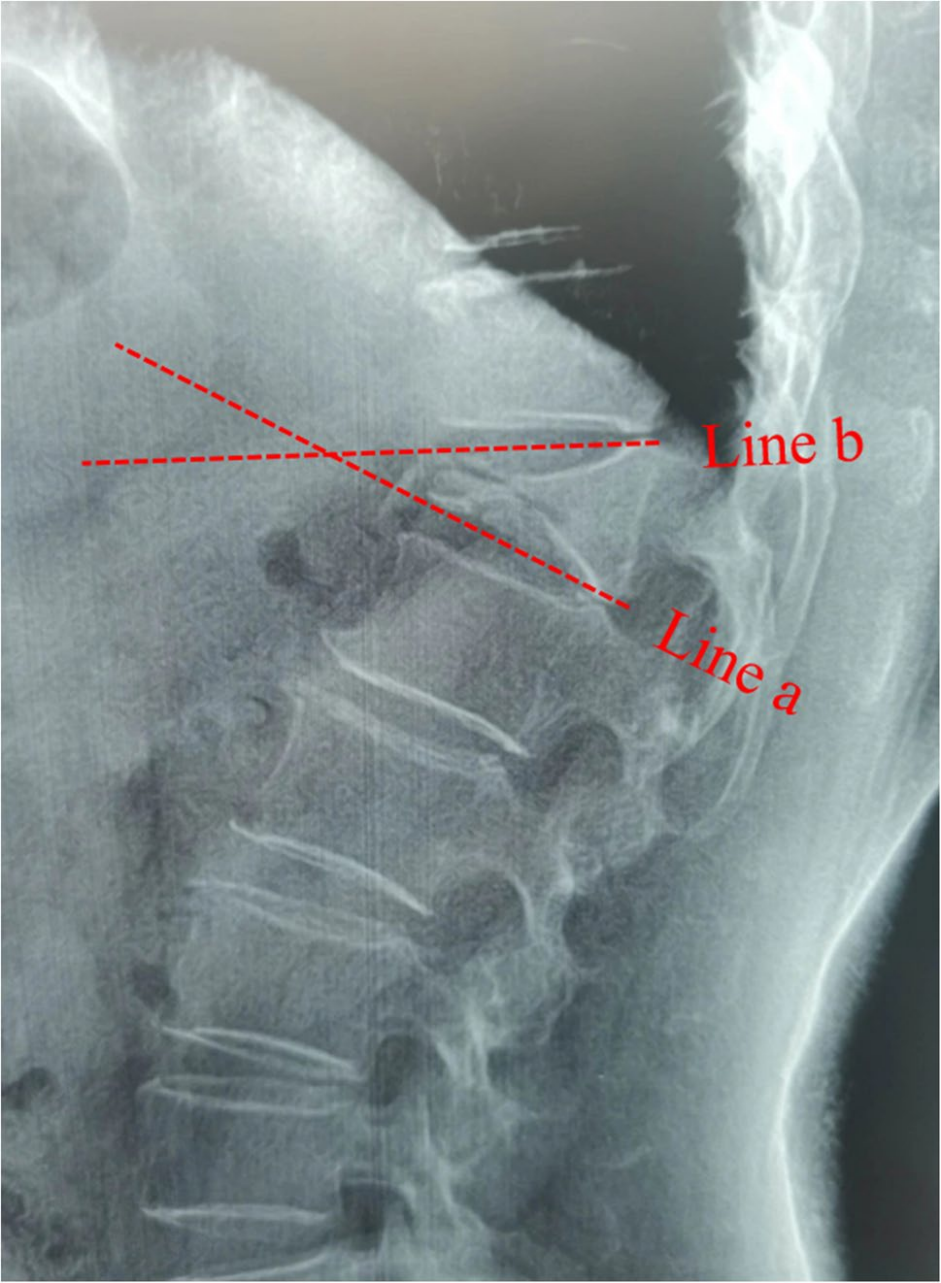
Measurement of the local kyphotic angle. Local kyphotic angle was defined as the angle formed by the upper endplates (line b) and lower endplates (line a) of the fractured vertebral body

**Fig. 2:**
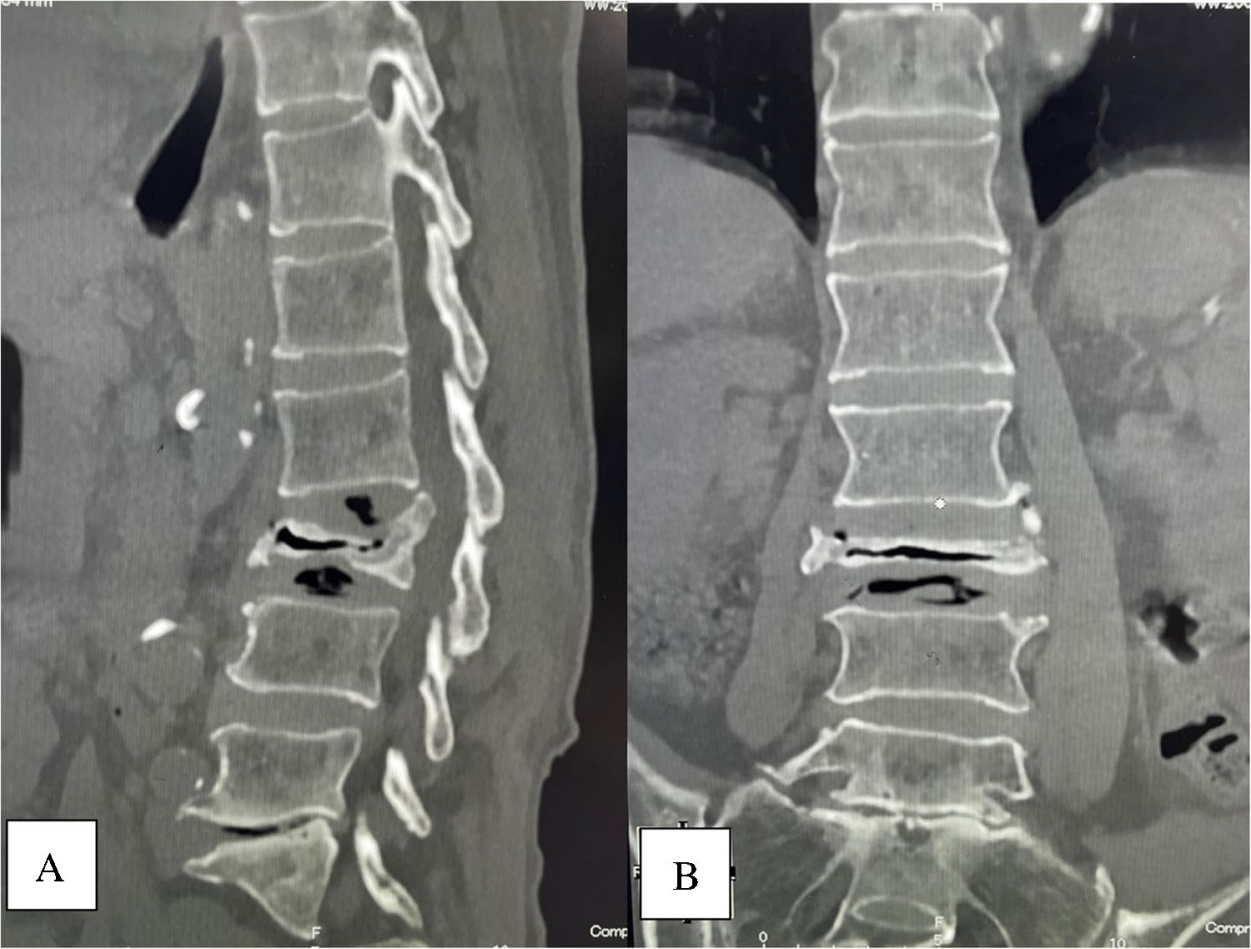
Intravertebral vacuum cleft was visible on sagittal (**A**) and coronal (**B**) views of computed tomography

BMI (kg/m^2^) was calculated as weight (kg) divided by the height squared (m^2^). In terms of injured vertebral segment, the thoracic junction, thoracolumbar junction, and lumbar junction were defined as T5–T9, T10–L2, and L3–L5, respectively. Vertebral height loss was calculated as the percentage of anterior vertebral body height relative to the average anterior vertebral body height of the upper and lower adjacent levels [4]. Local kyphotic angle was defined as the angle between the superior and inferior endplate of the fractured level (Fig. 1). The intravertebral vacuum cleft (IVC) sign was defined as a half-moon-shaped euphotic area located in the fractured vertebral body on plain radiography or a low-density area on computed tomography (CT) (Fig. 2). The definition of posterior fascia injury was based on the MRI finding of fascia edema and focal tenderness on physical examination in relation to the level of fascia edema [9].

**Fig. 3:**
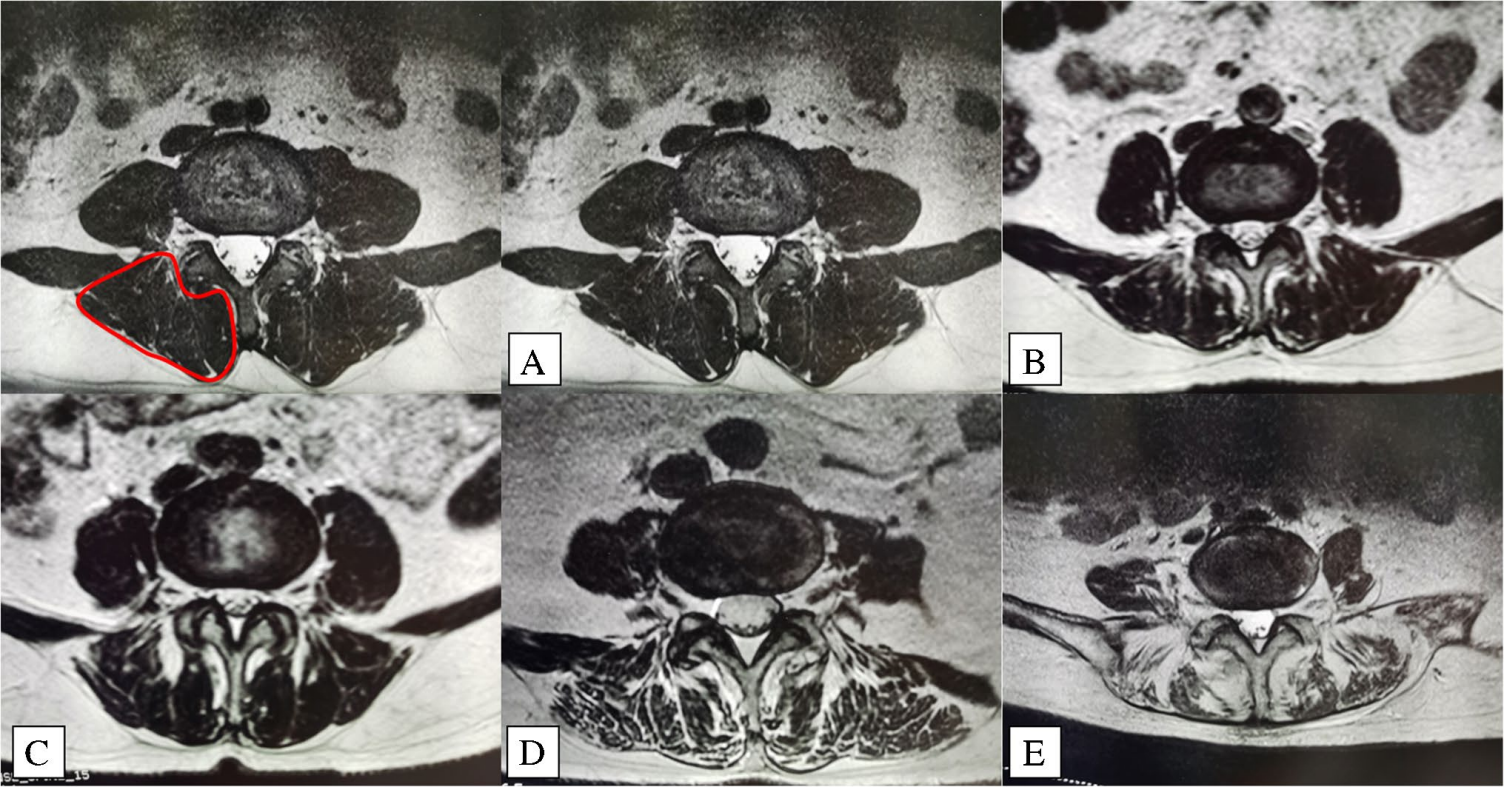
Goutallier classification. Fascial boundary of the paraspinal muscle (red line) on axial T2-weighted MRI. **A** Grade 0, normal, almost no fat infiltration in the muscle. **B** Grade 1, fatty streaks within the muscle. **C** Grade 2, fat infiltration was less than muscle mass. **D** Grade 3, fat infiltration was approximately equal to muscle mass. **E** Grade 4, fat infiltration was greater than muscle mass

The paravertebral muscles in this study were defined as multifidus muscle and erector spinae. Regarding paraspinal muscle fatty degeneration, two independent reviewers who were blinded to the clinical outcome scores utilized axial T2-weighted MRI to perform Goutallier classification system (GCS) of the bilateral paraspinal muscles at the L4 − 5 intervertebral disc level [12]. Patients were classified as grade 0 if there were no visible fat streaks in the muscle, grade 1 if there were minimal fatty streaks in the muscle, grade 2 if there was more muscle present than fat, grade 3 if fat and muscle were present in equal quantity, and grade 4 if more fat was present than muscle (Fig. 3). The two reviewer scores were then averaged to determine the final Goutallier grading.

The cross-sectional area (CSA) of the paraspinal muscle was evaluated using the region of interest (ROI). The functional cross-sectional area (fCSA) of the paraspinal muscle was measured using the threshold method. The fCSA referred to the fatfree, lean paraspinal muscle. Images were processed using ImageJ software (version 1.53, U.S. National Institutes of Health, Bethesda, MD, USA). Signal intensity was represented by the mean grayscale of the ROI quantified by the software, with higher scores indicating greater intensity. Wide oedema, haemorrhage, and inflammation were excluded in consideration of measurement bias. Finally, the results of the two observers were averaged. ROI-CSA and ROI-fCSA were recorded as the mean values of both muscle regions in each patient. The ratio of fCSA/CSA was analyzed, and it decreased as the muscles atrophied or more fat infiltrated them. Considering the wide differences among individuals, the ratio of CSA to disc CSA (D-CSA) was used to balance body size differences in the aforementioned parameters.

Vertebral height restoration was calculated as the percentage of the anterior vertebral body restoration height relative to the average anterior vertebral body height of the upper and lower adjacent levels. A satisfactory cement distribution was defined when the spongiform dispersive bone cement in contact with the upper and lower endplates, and an unsatisfactory cement distribution was defined when the bilateral cement masses were isolated and rarely connected to each other [1]. Facet joint violations were defined as needle trajectory contacts with joint surfaces or travel within the facet joint in postoperative CT images [13]. Cement leakage includes leakage in veins, discs, and the spinal canal.

### Functional assessment

For patients with residual low back pain, thoracic and lumbar MRI examinations were performed at the one year postoperative outpatient follow-up to detect a new fracture. The visual analog scale (VAS) was used to evaluate the RBP, with a score of 0 indicating no pain and 10 indicating the most severe pain. In this study, the patients were divided into RBP group (VAS score ≥ 4) and control group (VAS score < 4).

### Surgical procedures and management

All procedures were performed by senior physicians with PVA qualifications. The patient was in a prone position, and fluoroscopic positioning was performed by a C-arm X-ray machine. Local anaesthesia was performed on the articular capsule, the local muscle, and the fascia before diaplasis. The needle was inserted through unilateral or bilateral pedicle surface projection positioning of the surgical vertebrae. Then, the guide needle, dilation cannula, and work cannula were replaced sequentially under fluoroscopy. The C-arm X-ray machine fluoroscopy determined that the puncture needle exceeded the posterior edge of the vertebral body, and the needle tip did not cross the medial cortex. The needle core was pulled out after the satisfactory position was determined by lateral fluoroscopy. Surgical procedures for PVP patients: under the fluoroscopy of the C-arm X-ray machine, the pressure syringe was used to inject the high-viscosity or low-viscosity bone cement into the responsible vertebra. Surgical procedures for PKP patients: prior to injection of bone cement, a deflated balloon was placed into the vertebral body and inflated to restore the height of surgical vertebrae and the balloon was then deflated and withdrawn. In addition to using the balloon, the operation steps of PKP were similar to the previous method. Finally, the diffusion, distribution, displacement, and leakage of bone cement were observed under the fluoroscopy of the C-arm X-ray machine. After satisfactory, the injection device was removed, the wound was compressed to stop bleeding, and a sterile dressing was applied.

Patients were prescribed calcium tablets (600–1200 mg/day), calcitriol (0.25–0.5 μg/day), and alendronate (70 mg/ week) after surgery. Regarding pain management, patients who acquired adequate pain relief were not prescribed any analgesic drug, and those who still suffered from inadequate pain relief or pain obviously affecting basic daily life were prescribed non-steroidal anti-inflammatory drugs (NSAIDs); non-opioid analgesic drug was added if the NSAIDs were not effective.

### Statistical analyses

All data were analyzed with SPSS version 26 (IBM, Armonk, NY, USA). Quantitative data were presented as the mean ± standard deviation and compared with Student’s *t* test while categorical data were compared using the Fisher’s exact test and chi-square test. Multivariable analysis was performed using multiple logistic regression. Parameters were included in multivariable analysis based on clinical significance and statistical significance in univariable analysis. *P*-value < 0.05 was considered statistically significant.

## Results

### General information

In the present study, a total of 86 patients out of 876 patients with OVCF had poor postoperative back pain relief, and these patients were classified as the RBP group (VAS score ≥ 4). Mean age was 76.2 ± 8.4 years; 567 were female and 309 were male. The mean BMI was 23.8 ± 3.1 kg/m^2^ (range, 19.1–32.2 kg/m^2^). BMD with T-value ranging from − 2.5 to − 5.7 SD (mean, − 3.6 SD). The associated clinical and radiological characteristics are described in Table 1.

**Table 1:** Characteristics of patient group

Characteristic	Value
Number of patients	876
Age (years)	76.2 ± 8.4 (55–95)
Gender	
Female/male	567/309
BMI (kg/m^2^)	23.8 ± 3.1 (19.1–32.2)
Comorbidities	
Hypertension/diabetes/COPD/others	181/227/274/194
Preoperative VAS	7.4 ± 1.4 (5–9)
BMD (T score)	− 3.6 ± 1.2 (− 2.5 to − 5.7)
Injured vertebral segment	
Thoracic junction/thoracolumbar junction/lumbar junction	169/457/250

Abbreviations: *BMI*, body mass index; *COPD*, chronic obstructive pulmonary disease; *VAS*, visual analog scale; *BMD*, bone mineral density

### Univariate analysis

Comparisons of patient-independent variables between the RBP and control groups are summarized in Table 2. The comparison of results for the patients in the RBP group and the control group indicated that there were no significant differences between the two groups regarding age, gender, BMI, comorbidities, preoperative VAS, injured vertebral segment, vertebral height loss, local kyphosis angle, surgical method, surgical approach, cement viscosity, cement volume, vertebral height restoration, cement leakage, or postoperative bracing treatment (*P* > 0.05). With respect to paraspinal muscle fatty degeneration, there were no statistically significant differences in CSA and CSA/D-CSA (*P* > 0.05), but when comparing the data in the Goutallier grading, fCSA and fCSA/CSA, the difference was significant (*P* < 0.001). The results also showed that the following factors were significantly different between the control and back pain groups: BMD (*P* < 0.001), intravertebral vacuum cleft (*P* = 0.005), posterior fascia injury (*P* < 0.001), cement distribution (*P* = 0.004), and facet joint violations (*P* < 0.001). Collectively, these data indicated that the above factors were potentially associated with postoperative back pain and were included in the multivariate analysis.

**Table 2:** Univariate analysis for the risk factors of RBP after PVA

Variable	RBP group (86)	Control group (790)	*P* value
Patients characteristics			
Age (years)	77.4 ± 8.4	75.9 ± 7.6	0.086
Gender (female/male, *n*)	52/34	515/275	0.384
BMI (kg/m^2^)	25.6 ± 5.5	25.3 ± 4.7	0.627
Comorbidities			0.735
Hypertension	17	164	
Diabetes	22	205	
COPD	31	243	
Others	16	178	
Preoperative VAS	7.6 ± 2.2	7.3 ± 1.6	0.222
Preoperative imaging data			
BMD (T score)	− 3.2 ± 0.1	− 2.9 ± 0.3	< 0.001
Injured vertebral segment			0.900
Thoracic junction	15	154	
Thoracolumbar junction	46	411	
Lumbar junction	25	225	
Vertebral height loss (mm)	31.2 ± 10.3	28.9 ± 10.9	0.062
Local kyphosis angle (°)	26.9 ± 7.2	25.5 ± 4.8	0.082
Intravertebral vacuum cleft			0.005
No	62	664	
Yes	24	126	
Posterior fascia injury			< 0.001
No	23	461	
Yes	63	329	
Paraspinal muscle fatty degeneration			
Goutallier grading			< 0.001
Goutallier grade 0–2	20	535	
Goutallier grade 3–4	66	255	
CSA (mm^2^)	2064.8 ± 346.7	1993.9 ± 329.8	0.060
fCSA (mm^2^)	1172.0 ± 427.6	1647.6 ± 232.2	< 0.001
fCSA/CSA (%)	38.6 ± 12.3	32.3 ± 8.4	< 0.001
CSA/D-CSA (%)	153.1 ± 27.4	158.9 ± 19.2	0.059
Surgical data			
Surgical method			0.416
PVP	19	209	
PKP	67	591	
Surgical approach			0.238
Unilateral	70	598	
Bilateral	16	192	
Cement viscosity			
High	55	458	0.285
Low	31	332	
Cement volume(ml)	3.4 ± 1.1	3.5 ± 1.3	0.492
Postoperative radiological parameters			
Vertebral height restoration (%)	14.7 ± 5.5	15.5 ± 6.1	0.244
Cement distribution			0.004
Satisfactory	30	404	
Unsatisfactory	56	386	
Facet joint violation			< 0.001
No	36	602	
Yes	50	188	
Cement leakage			0.529
No	56	487	
Yes	30	303	
Bracing treatment			0.441
No	40	402	
Yes	46	388	

Abbreviations: *BMI*, body mass index; *COPD*, chronic obstructive pulmonary disease; *VAS*, visual analog scale; *BMD*, bone mineral density; *CSA*, cross-sectional area; *fCSA*, functional cross-sectional area; *D-CSA*, disc cross-sectional area; *PVP*, percutaneous vertebroplasty; *PKP*, percutaneous kyphoplasty

### Multivariate analysis

Multivariate logistical regression analysis was performed to detect independent risk factors for RBP. As shown in Table 3, posterior fascia injury (odds ratio (OR) = 5.23; 95% confidence interval (CI) 3.12–5.50; *P* < 0.001), as regards paraspinal muscle fatty degeneration, including Goutallier grading (*OR* = 12.23; 95% *CI* 7.81–23.41; *P* < 0.001), fCSA (*OR* = 3.06; 95% *CI* 1.63–6.84; *P* = 0.002), fCSA/CSA (%) (*OR* = 14.38; 95% *CI* 8.80–26.29; *P* < 0.001), and facet joint violation (*OR* = 8.54; 95% *CI* 6.35–15.71; *P* < 0.001) were identified as independent risk factors for RBP.

**Table 3:** Multivariate logistic regression analysis for the risk factors of RBP after PVA

Characteristic	*OR* value	95% *CI*	*P* value
BMD (T score)	3.56	0.79–4.12	0.109
Posterior fascia injury	5.23	3.12–5.50	< 0.001
Intravertebral vacuum cleft	1.22	0.71–1.79	0.089
Goutallier grading	12.23	7.81–23.41	< 0.001
fCSA (mm^2^)	3.06	1.63–6.84	0.022
fCSA/CSA (%)	14.38	8.80–26.29	< 0.001
Cement distribution	1.54	0.84–1.79	0.413
Facet joint violation	8.54	6.35–15.71	< 0.001

## Discussion

Although PVA provides quick pain relief, improved physical function, and good restoration of vertebral height, some patients still experience considerable residual back pain. In addition, although sarcopenia is known to compromise quality of life and increase the risks of falls and disability in elderly patients, there has been few studies on how paraspinal muscle fatty degeneration contributes to poor clinical outcome after PVA. Therefore, we deeply investigated whether paraspinal muscle fatty degeneration is an independent risk factor of RBP following PVA. The result demonstrated that posterior fascia injury (odds ratio (OR) = 5.23; 95% confidence interval (CI) 3.12–5.50; *P* < 0.001), as regards paraspinal muscle fatty degeneration, including Goutallier grading (*OR* = 12.23; 95% *CI* 7.81–23.41; *P* < 0.001), fCSA (*OR* = 3.06; 95% *CI* 1.63–6.84; *P* = 0.002), fCSA/CSA (%) (*OR* = 14.38; 95% *CI* 8.80–26.29; *P* < 0.001), and facet joint violation (*OR* = 8.54; 95% *CI* 6.35–15.71; *P* < 0.001) has a more significant impact on RBP.

In our study, posterior fascia injury was found to be an independent risk factor for RBP. Yan et al. [9] determined through a prospective cohort study that thoracolumbar fascia injury was closely related to residual back pain after percutaneous vertebroplasty. In a retrospective analysis study reported by Yang et al. [10], thoracolumbar fascia injury is a strong risk factor for unsatisfactory back pain relief after PVP. One possible mechanism is that pain originating from the micromovement of the vertebral fracture can be prevented due to the elimination of microfractures after PVA, while soft-tissue injury could be an alternative cause of RBP. Another possible mechanism is that it is a counterbalancing injury. Due to the anterior compression of the vertebral body, the increased bending moment must be counterbalanced by the posterior structures such as muscles, ligaments, and posterior fascia. In addition, one plausible explanation for this result is that the thoracolumbar fascia is rich in nerve endings [8].

The finding that paraspinal muscle fatty degeneration is a risk factor for RBP after PVA has rarely been mentioned in previous studies. This study investigated the effect of quantitative analysis and fatty degeneration of the paraspinal muscle on the residual back pain, in addition to the risk factors for poor efficacy described in previous studies. The paraspinal muscles play a critical role in the stability and functional movement of the spine [14]. Habibi et al. [15] reported that fatty degeneration of the paraspinal muscle in the thoracolumbar region is strongly correlated with the occurrence of new OVCFs. Katsu et al. [16] reported that a larger relative CSA of the multifidus and erector muscles may play a more significant role in fracture union for patients with OVCF than in those with fatty degeneration. In our study, smaller fCSA and larger fCSA/CSA of the paravertebral muscles may play a more important role in the residual pain group compared to the control group. However, the total CSA of the paraspinal muscle and CSA/D-CSA did not affect VAS score, suggesting that lean muscle is more important than total muscle mass for pain relief. Multivariate regression analysis proved that paraspinal muscle degeneration was an independent risk factor for postoperative residual back pain. Muscle fatty degeneration can occur without a change in total muscle size and is an important indicator of the quality and functional status of the muscle. Bo et al. [5] also suggested that sarcopenia in OVCF patients after PVP were more prone to residual back pain. Given the growing body of evidence suggesting that paraspinal muscle fatty degeneration is a manifestation of musculoskeletal frailty, we would recommend that sarcopenia be assessed opportunistically on preoperative MRI scan to identify patients that may benefit from additional preoperative optimization. For those patients with OVCF whose MRI shows severe paraspinal muscle fatty degeneration, in addition to the treatment of fracture, paraspinal muscle sarcopenia also needs to be intervened and treated at the same time. Moreover, combined treatment with rehabilitation medicine and nutrition department can be carried out during the perioperative period, which may include nutrition optimization, preoperative physical therapy, or other medical therapy. In the process of rehabilitation exercise, physicians can also guide these patients to perform muscle function exercises to increase muscle strength.

Moreover, multivariate analysis of this study showed that facet joint violation was another risk factor for postoperative pain. Lin et al. [17] found that facet joint violation contributed to the development of residual low back pain after PVA surgery and proposed that the incidence of facet joint violation was strongly correlated with the proficiency of surgical techniques. Li et al. [8] also identified facet joint invasion as an independent risk factor for postoperative low back pain, which is consistent with our results. The reason for this may be that the facet joint and capsule ligament are full of mechanoreceptors and pain-detecting nociceptors, which are susceptible to direct mechanical impairment. Another explanation for this result is related to the deleterious effects of facet joint violations on secondary inflammatory reactions and the presence of osteoarthritis.

The limitations of the present study mainly include the following items: first, our study is a retrospective single-center study with a small number of cases, and the data contained do not record the location and severity of bone cement leakage. Therefore, a prospective, multicenter study with a larger sample size is needed to verify the conclusions of this study. Second, the patients were divided into two groups based on VAS scores at one year of follow-up and did not compare differences during follow-up. Nevertheless, several risk factors for residual back pain were confirmed, at least in part, especially highlighting the importance of paraspinal muscle fatty degeneration and they will be important for improving surgical efficiency.

In conclusion, posterior fascia injury, paraspinal muscle fatty degeneration, and facet joint violation were identified as independent risk factors for RBP, with paraspinal muscle fatty degeneration playing an important role.
